# Exposure and posttraumatic stress symptoms among first responders working in proximity to the terror sites in Norway on July 22, 2011 – a cross-sectional study

**DOI:** 10.1186/s13049-015-0104-4

**Published:** 2015-02-24

**Authors:** Laila Skogstad, Anja M Fjetland, Øivind Ekeberg

**Affiliations:** Department of Acute Medicine, Oslo University Hospital, Ulleval, Box 4956, Nydalen, 0424 Oslo Norway; Department of Behavioural Sciences in Medicine, Institute of Basic Medical Sciences, Faculty of Medicine, University of Oslo, Oslo, Norway

**Keywords:** Disaster, First responders, Mental distress, Posttraumatic stress, Terrorism

## Abstract

**Background:**

Norway experienced two terror attacks on July 22, 2011. A car bomb exploded in the Oslo government district killing eight people. Shortly after, 69 adolescents gathered at a political youth camp were shot and killed at Utøya Island. First responders were exposed to multiple risk factors for the development of posttraumatic stress symptoms (PTSS).

**Methods:**

This cross-sectional study investigated the degree of perceived peritraumatic strain among police officers, fire-fighters, and ambulance personnel, as well as the prevalence and predictors of PTSS. A questionnaire was completed by 89 ambulance personnel, 73 fire-fighters, and 76 police officers working close to the terror sites, 8–11 months after the event. PTSS were assessed using the PTSD Check List (PCL-S).

**Results:**

Merging all groups, 68% reported to have witnessed injured/dead people, but only 5.7% reported this as very/extremely strainful. The PCL-S scores were low and not significantly different among the three professions (*Median* = 19-20, range 17-64). The prevalence of possible PTSD (cut-off > 50) was 1.3 %, and 2 % had scores indicating sub-threshold PTSD. Dissociation predicted higher PTSS-level in all groups (β 1.6-5.1), witnessing injured/dead among ambulance personnel (β 2.5) and feeling overwhelmed among police officers (β 1.2).

**Conclusion:**

First responders were exposed to deaths, injuries, and destruction, but few reported this as highly stressful. The prevalence of possible PTSD was low in all occupational groups, and symptoms of dissociation were found to be the most important predictor.

## Background

On July 22, 2011, there were two terror attacks in Norway caused by a single perpetrator. A bomb was detonated in the government district of Oslo. Eight people died, many were injured. A few hours later, shooting was reported from Utøya Island. During the second attack 69 young adults and teenagers, participating at the Norwegian Labour Party’s summer youth camp, were shot and killed. Many were injured [1-3]. At Utøya Island, there was no possibility of escape other than swimming, and there were few places to hide, which turned the island into a “trap” where shootings took place for about 1.5 hours. All emergency personnel in the areas were mobilized immediately. This was the most extensive terror act in Norway since the Second World War, and the events were perceived as an assault on freedom of speech in targeting the government and a political party.

Terror attacks affect the victims and their families, but the rescuers also experience extensive damage, death, and casualties. Sometimes, they even put their own lives and health in danger. These events impose unfamiliar and heavy demands, in particular on the first responders.

Victims of traumatic events have an increased risk of developing posttraumatic stress symptoms (PTSS) or posttraumatic stress disorder (PTSD). The diagnosis of PTSD requires a severe traumatic event. Rescue workers experience traumatic events repeatedly and report more current PTSD (10%) than the general population (3.5%) [4]. Among the different groups of rescue workers, there is a higher reported prevalence of PTSD in ambulance personnel [4].

Many traumatic events can lead to PTSD, in particular prolonged trauma, events caused by human evil (i.e., terrorism) [5], and events involving children [6]. There are divergent findings about specific risk factors for rescue workers, although high level of exposure to the traumatic event (e.g., working close to the site), assisting survivors or close contact with the victims and shortage of supplies and resources [6] are predisposing. Intense fear, lack of control, peritraumatic dissociation, and perceived personal threat, are additional risk factors. After the event, lack of social support [7] and intercurrent life stress [8] are variables that predict PTSD.

Occupational stress [9] is important, but there is disagreement about whether previous experience with disasters or repeated traumatic experiences [6,10], longer job experience/training, and age [4,5,8] are risk factors or protective factors. In contrast to studies of civilians, there is no difference in the prevalence of PTSD between female and male rescue workers [11].

As terror attacks are seldom in most Western countries, when it happens, rescue workers face unfamiliar tasks, more destruction and danger than they are used to. As several rescue workers develop posttraumatic problems after terror attacks, it is important to study the rather few serious terror attacks that are faced.

Thus, the main aims of the present study were to investigate the degree of perceived peritraumatic strain as well as the prevalence and predictors of PTSS among first responders (police officers, fire-fighters, and ambulance personnel) working close to the sites of the terror attacks.

## Methods

This cross-sectional study investigated personnel involved in the rescue operation after the terror attacks in Norway on July 22, 2011. The questionnaire included background variables, contribution, and experiences during the rescue operations, and how the event affected them. They were distributed between March and June 2012, approximately 8 – 11 months after the terror attacks (mean = 10 months). A reminder was sent one month after the first request. The study was anonymous and the questionnaire was distributed with an information letter. The return of the questionnaire was assumed to imply informed consent.

### Material

Since working in proximity to the disaster site has been found to predict PTSS, professional first responder such as ambulance personnel (including nurses, physicians, and helicopter emergency medical service working at the site of terror), fire-fighters and police officers working either in the government district, at Utøya Island or both were included. These subgroups were selected from the total population (see flow chart).

The National Police Directorate included personnel from Oslo, Asker/Baerum, and the northern district of Buskerud. Fire-fighters from 10 independent units in four counties, and ambulance personnel from 18 units in six counties participated. A leader from each unit was contacted. The leaders were responsible for the distribution and collection of the questionnaires.

**Figure 1:**
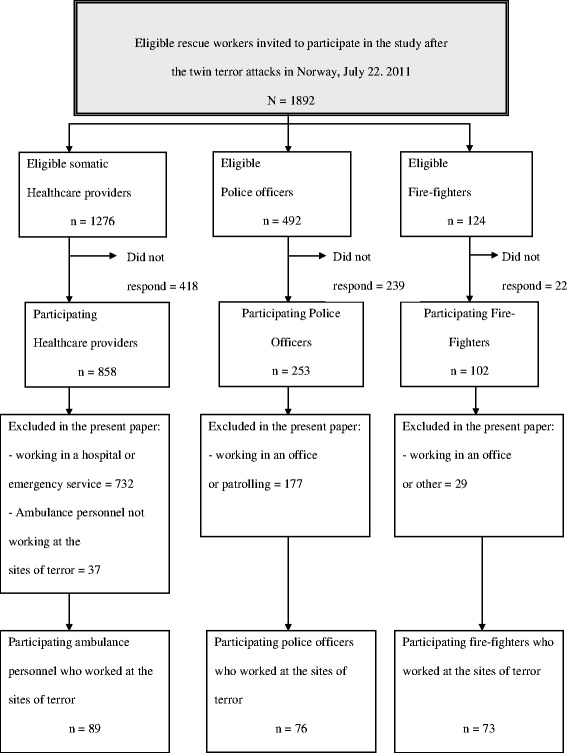
**Eligible rescue workers invited to participate in the study after the twin terror attacks in Norway, July 22. 2011.** N = 1892.

Of those responding in the main study, 89/126 (71%) of the ambulance personnel worked close to the site of terror. The corresponding numbers for police was 76/253 (30%) and for fire-fighters 73/102 (72%). The majority of the police officers had other obligations, like securing other possible terror targets, investigation and research operations. Figure 1 shows the flow chart.

### Assessments

Most of the items included in the questionnaire were developed by The Norwegian Centre for Violence and Stress Studies and used in a cross-sectional study of the Norwegian personnel mobilized during the 2004 tsunami disaster [12]. A replica of that study, thus, made it possible to compare data from two samples of Norwegian rescue workers.

Sociodemographic characteristics were collected in terms of age, sex, the working sites and tasks during the rescue operation. Most of these data are summarized in Table 1.

**Table 1:** **Background characteristics, work experience, training and resources**

***N*** **= 238,** ***n*** **(%)**	***Police officers n = 76***	***Fire-fighters n = 73***	***Ambulance personnel n = 89***	***p-value***
**Gender**				< .001**
Male	56 (75)	71 (99)	64 (72)	
**Age**				.009*
<30 years	15 (20)	7 (10)	29 (33)	
30–49 years	51 (68)	52 (71)	49 (55)	
>50 years	9 (12)	14 (19)	11 (12)	
**Work experience in current organization**				.006*
<1 year	6 (8)	1 (1)	5 (6)	
1–5 years	26 (35)	13 (18)	35 (39)	
>5 years	43 (57)	59 (81)	49 (55)	
**Training yes**				
Work experience in similar tasks	55 (72)	54 (74)	54 (62)	ns
Training on similar tasks	64 (84)	55 (75)	66 (76)	ns
Disaster drill	49 (64)	47 (64)	79 (90)	< .001**
Experience of previous incident with > 5 casualties	15 (20)	20 (27)	29 (33)	ns
Sufficient resources. Scale 1-5	2.8 (2.6-3.1)	3.7 (3.5-3.9)	3.7 (3.5-4.0)	< .001**

Note: *p < .05, **p < .001.

**Figure 2:**
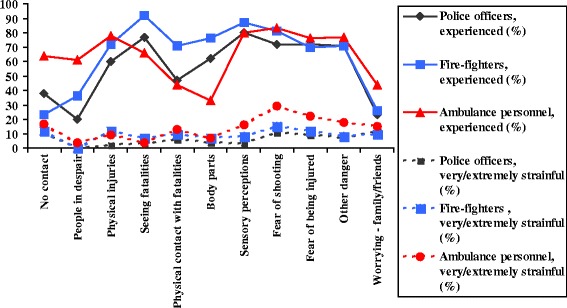
**Peritraumatic exposure and perceived threat.**

Witnessing was measured using seven items: witnessing disaster victims (1) searching for next of kin; (2) in despair at the campsite; (3) with major physical injuries; (4) dead bodies; (5) physical contact with dead bodies; (6) body parts; and (7) strong smells or other sensory perceptions. A two-factor solution was found: witnessing a) people in despair (item 1-2); b) people with major injuries/fatalities (item 3-7). All items were dichotomized (no/yes). The respondents also scored each item to what degree the stressor were perceived as strainful (*not/mildly /moderately, and very/extremely*) (Figure 2).

Perceived threats were assessed with four self-designed items. Whether subjects experienced: (1) fear of explosion/shooting; (2) fear of being injured; (3) other risks/uncertainty; and (4) concern for relatives/friends who might had been at the terror sites during the attacks. The response alternatives for all items were: 0 = *no, not experienced*; 1 = *yes, but not strainful*; 2 = *yes, moderately strainful*; and 3 *yes, very strainful* (Figure 2). Items 1-3 were summarized and named peritraumatic threat. The Cronbach’s Alpha was .87.

### Resources

The variable “We had sufficient resources to carry out satisfying work” was measured on a Likert scale 1-5, 1 = *not at all*, 5 = *to a very high degree*. Results are reported with a median split score: 1-3 *(to a low degree)* and >3 *(to a high degree)*.

Peritraumatic dissociation and arousal during the rescue operations were assessed using eight items: (1) a feeling of “numbness”; (2) a feeling of not being aware of the surroundings; (3) a feeling that what you experienced was not real; (4) a feeling of not being yourself; (5) not remembering what happened, or only parts of it; (6) a feeling of sharpened attention; (7) reduced need for sleep and/or rest; and (8) positive activation (more energy or an intense sense of coping). A five-point Likert scale was used: 1 = *not at all* and 5 = *to a very high degree* (Table 2). A factor analysis revealed two constructs; dissociation and arousal. Items 1-5 measure dissociation, and items 6-8 measure arousal. Cronbach’s Alpha was .78 for dissociation and .76 for arousal.

**Table 2:** **Psychological responses**

**Mean (95% confidence interval)**	***Police officers n = 76***	***Fire-fighters n = 73***	***Ambulance personnel n = 89***	***p value***
1) Feeling of numbness	1.5 (1.3-1.6)	1.6 (1.4-1.8)	1.9 (1.7-2.1)	< .05*
2) Not being aware of the surroundings	1.3 (1.2-1.4)	1.4 (1.2-1.6)	1.6 (1.4-1.8)	< .05*
3) Sense of unreality	2.2 (1.9-2.4)	2.9 (2.6-3.2)	2.7 (2.5-3.0)	< .01*
4) Feeling of not being “yourself”	1.3 (1.1-1.5)	1.3 (1.2-1.5)	1.5 (1.3-1.7)	*ns*
5) Did not remember what happened or parts of the event	1.6 (1.3-1.9)	1.3 (1.2-1.5)	1.5 (1.3-1.7)	*ns*
6) Sharpened attention	3.8 (3.5-4.0)	3.4 (3.1-3.6)	3.7 (3.4-3.9)	< .05*
7) Reduced need for sleep/rest	3.3 (3.0-3.6)	2.1 (1.8-2.4)	2.7 (2.4-3.0)	< .001**
8) Positive activation (more energy/intense sense of coping)	3.2 (2.9-3.4)	2.6 (2.4-2.9)	2.9 (2.7-3.2)	< .05*
9) Overwhelmed/helpless	2.6 (2.3-2.8)	2.6 (2.4-2.8)	2.9 (2.7-3.2)	*ns*
10) Lack of control	2.5 (2.2-2.8)	2.2 (2.0-2.5)	2.7 (2.4-2.9)	< .05*

*Note.* Dissociative symptoms (1-5), arousal (6-8), and lack of coping (9-10).

(Scale: 1 = not at all and 5 = to a very high degree).

*p < .05, **p < .001.

### Psychological responses

Two questions assessed perceived psychological responses: (a) did you feel overwhelmed; and (b) did you feel that you had no control? These items were scored on a five-point Likert scale: 1 = *not at all* and 5 = *to a very high degree* (Table 2). Posttraumatic stress symptoms. The PTSD Checklist (PCL-S) [13], a widely used self-reported measure of PTSD, screened for PTSD symptoms [14]. Seventeen items assess the full domain of PTSD symptoms based on the Diagnostic and Statistical Manual of Mental Disorders Fourth edition (DSM-IV) [15]. Each item was scored on a five-point Likert scale (1 = *not at all*, to 5 = *very often*) where the overall scores ranged from 17 to 85. A score of 31 – 38 seems to be a common cut-off level to identify most PTSS cases [16,17] and a cut-off score of 50 has been used as an indicator of PTSD (Table 3). In the present sample the Cronbach’ Alpha was .91.

**Table 3:** **Posttraumatic stress symptoms**

**Median (range) or** ***n*** **(%)**	***Police officers n = 76***	***Fire-fighters n = 73***	***Ambulance personnel n = 89***	***p value***
PCL-S total score	19 (17-36)	19 (17-64)	20 (17-64)	*ns*
Score ≥ 50 possible PTSD case	0 (0.0)	2 (2.7)	1 (1.1)	*ns*
Score 35 - 49 possible sub-threshold PTSD	1 (1.3)	1 (1.4)	3 (3.4)	*ns*

Note: *PTSD Checklist- specific version (PCL-S), range = 17- 85.*

### Statistical analysis

The data were presented as means with 95% confidence intervals, or percentages. In general there were few missing data (0.4 – 1.7%). The witnessing items had a slightly higher percentage of missing data (1.9 - 6.7%) among responders. Where appropriate, the variables were dichotomized. Chi-squared and Kruskal–Wallis tests were used to compare proportions, and ANOVA was used to compare means. Linear regression analysis identified the predictors of PTSS. Each occupational group was analyzed separately. In the first part of the linear regression analysis each variable was univariately tested with the continuous PCL score (entry .05, and removal 1.0). Variables with a *p*-value < .05 were then included in the multivariate analyses (stepwise). Each group was tested separately in the multivariable analysis as well. SPSS (version 18.0, SPSS, Chicago, Il) was used.

### Ethics

This study was anonymous, and approval from the Regional Ethics Committee was not required. Oslo University Hospital’s Privacy Protection Supervisor approved the study. The data were stored on the research server at the hospital.

## Results

Most of the police officers worked in the Oslo government district whereas the fire-fighters were located mainly at Utøya Island. Ambulance personnel worked either in Oslo, at Utøya Island, or at both sites (28%). Most of the first responders had over five years of work experience (police = 57%, fire-fighters = 81%, and ambulance personnel = 55%). The fire-fighters had significantly more work experience (χ^2^ = 14.5, *p* < .01) and were significantly older than the other groups (χ^2^ = 13.6, *p* < .01). Some had previous experience of working in incidents where five or more fatalities occurred (ambulance personnel = 33%, police = 20%, and fire-fighters = 27%, n.s.). The majority had previously trained on similar tasks (ambulance personnel, 76% vs. police, 84% vs. fire-fighters, 75%, n.s). A significantly higher number of ambulance personnel had participated in a disaster drill compared to the other groups (90% vs 64% vs 64%, *p* < .001).

The first responders mainly performed work in which they were trained. The police were securing the sites, taking care of spectators, and searching for survivors and dead. The fire-fighters were securing the areas where the dead bodies were collected, searching for survivors and dead, taking care of injured people, and transportation, particularly of dead people. The ambulance personnel mainly performed first aid, triage assessments, and transport to hospital. Police officers reported to have fewer resources to perform their rescue work (mean: police 2.8 vs. fire-fighters 3.7 vs. ambulance personnel 3.7, *p* < .001).

### Peritraumatic exposure and perceived threat

In Figure 2 peritraumatic exposure and perceived threat are presented. The fire-fighters often witnessed and had physical contact with dead and loose body parts, whereas the ambulance personnel more often witnessed people searching for next of kin and people in despair (items 1 – 6). Strong sensory perceptions (item 7) were experienced by > 80% (n.s. between groups). Threats (items 8 – 10) such as fear of shooting and being injured, were experienced by > 70 % (n.s. between groups). Worrying about next of kin (item 11) was more common in ambulance personnel (ambulance personnel = 44%, police = 23%, fire-fighters = 26%, *p* < .05). There were no statistically significant differences among the proportions who reported the items as “*very/extremely strainful*.

### Dissociative symptoms, arousal, and control

The levels of peritraumatic dissociation (Table 2, items a – e) were generally low, except moderate scores for sense of unreality (police, *M* = 2.2 vs. fire-fighters, *M* = 2.9 vs. ambulance personnel *M* = 2.7, *p* > .01). Significantly higher scores related to the arousal items (items f – h) were found among police officers for the item “reduced need for sleep and rest” compared with the fire-fighters (*p* < .001). The ambulance personnel had intermediate scores. All groups reported clearly sharpened attention (police, *M* = 3.8 vs. fire-fighters, *M* = 3.4 vs. ambulance personnel, *M* = 3.7, *p* < .05). The police officers reported more arousal than the fire-fighters (*M* = 3.2 vs. *M* = 2.6, *p* < .05). The fire-fighters reported somewhat lower levels for experience of lack of control compared with police officers and the ambulance personnel, although the levels were moderate.

### Posttraumatic stress symptoms at follow-Up

The PTSS levels (Table 3) were moderate with median PCL-S scores of 19 (range 17-36) for police, 19 (17-64) for fire-fighters, and 20 (17-64) for ambulance personnel (n.s.). No police officers, two fire-fighters (2.7%), and one ambulance worker (1.1%) scored above the cut-off score of 50, i.e. at a symptom level of possible PTSD. In addition, one police officer (1.3%), one fire-fighter (1.4%) and three ambulance personnel (3.4%), had sub-threshold scores (PCL-S = 35 – 50).

### Predictors for PTSS

As shown in Table 4, there were several univariate predictors for PTSS in all groups. The multivariate analyses (Table 5) showed that symptoms of dissociation were a significant independent predictor in all groups. Seeing injured people was a predictor for PTSS in ambulance personnel, and feeling overwhelmed was a predictor for PTSS in police officers. We have also run the analyses with all groups together, showing that the independent predictors were dissociation and feeling overwhelmed. By analyzing the three groups separately, we found that the predictors differed according to group.

**Table 4:** **Factors predicting a higher posttraumatic stress score univariate/unadjusted**

	***Police officers***		***Fire-fighters***		***Ambulance personnel***	
	**β**	***p*** **value**	**β**	***p*** **value**	**β**	***p*** **value**
Age	−1.6	< .05*		*ns*	– 2.5	< .05*
Gender, male = 1, female = 2		*ns*		*ns*	4.3	< .05*
Previous training, no/yes		*ns*		*ns*		*ns*
Work experience in similar tasks, no/yes		*ns*		*ns*		*ns*
Disaster drill, no/yes	−1.9	< .05*		*.065*		*ns*
Concerned about next of kin		*ns*		*ns*		*ns*
Witnessing injured/dead no/yes	1.6	< .05*		*ns*	3.7	<. 001**
Witnessing despaired people no/yes	1.8	< .05*		*ns*		*.074*
Reject victims, no/yes		*ns*		*ns*		*ns*
Lack of control (1-5)	1.1	< .05*		*ns*	2.1	<. 001**
Overwhelmed (1-5)	1.6	< .001**	2.0	< .05*	1.8	< .05*
Arousal (1-5)				*ns*	*1.6*	< .05*
Dissociation (1-5)	2.6	<.001**	4.7	< .001**	4.6	<. 001**

*Note*. Univariable linear Regression Analysis, stepwise. *p < .05, **p < .001.

**Table 5:** **Factors predicting a higher posttraumatic stress score multivariate/adjusted**

	***Ambulance personnel***				***Police officers***				***Fire-fighters***			
	**β**	**95% CI**	***t***	***p*** **value**	**β**	**95% CI**	***t***	***p*** **value**	**Beta**	**95% CI**	***t***	***p*** **value**
Witnessing injured/dead no/yes	2.5	0.7-4.3	2.8	< .05*								
Overwhelmed (1-5)					1.2	0.4-2.0	3.1	< .05*				
Dissociation (1-5)	4.2	2.8-5.7	5.8	< .05*	1.6	0.1-3.1	2.1	< .05*	5.1	2.4-7.7	3.8	< .001**

*Note*. Linear regression analysis, stepwise CI: confidence interval. *p < .05, **p < .001.

## Discussion

The main finding of the present study was that the first responders working close to the terror sites reported a low level of PTSS 10 months after the terror attack, and that there were both similar and different predictors of PTSS in the three occupational groups.

### Perceived peritraumatic strain

Ambulance personnel, police officers and fire-fighters working close to the sites of terror were exposed to highly traumatic experiences, both in terms of peritraumatic witnessing and perceived threat. Ambulance personnel more often reported witnessing disaster victims searching for next of kin and victims in despair, while fire-fighters more often reported witnessing loose body parts, dead bodies and to have had physical contact with dead people. Over 80% in all groups reported to have had strong sensory perceptions, and among ambulance personnel 13% scored this as very/extremely strainful. In addition, more than 70% reported peritraumatic threat in terms of fear of shooting, being injured or other risks. Despite witnessing potentially traumatic situations and perceiving peritraumatic threat, few reported this as very/extremely strainful, but more often by ambulance personnel.

After the tsunami in 2004, witnessing experiences were assessed in a study of Norwegian personnel working in the disaster-area [12]. More first responders working after the terror attacks in Norway reported witness experiences compared to personnel working after the tsunami. Even so, they more seldom assessed the experience as very/extremely strainful. The tsunami group was more heterogenic, including police, health care personnel but also journalists, travel agency personnel etc., some of these were probably not trained for working in disaster areas. They also worked in unfamiliar circumstances in a foreign country. Even though perceived peritraumatic strain was experienced by a substantial proportion of the first responders after the terror attacks, few reported this as very/extremely strainful, implying an ability to stay focused during the rescue work. This may be a result of previous training and work experience, and perhaps working in more familiar surroundings and with known colleges.

Previous studies have shown that repeated exposure may be a risk factor for PTSD [7]. Norwegian first responders are exposed to trauma during their everyday work, but they might be exposed to less severe events compared with some of their colleagues in other countries. The attacks on July 22, 2011 had a short duration and did not involve persistent threats for many hours or days. The person responsible for the acts of terror was arrested during the rescue operation.

### Prevalence of PTSS

First responders were exposed to highly traumatic experiences, but reported a low prevalence of possible PTSD (1.3%), and with no significant difference between the three groups. These results agree well with the findings after the tsunami in 2004 [12] and with those reported after the terror attack in Madrid [18]. The latter study reported a 1.3% prevalence of PTSD in police officers two months after the attack. In contrast, after the World Trade Centre (WTC) attack, 11% of the rescue-, recovery-, and clean-up workers were considered to suffer from PTSD [19], a similar prevalence was found after the Oklahoma bombing (13%) [20]. Somewhat lower prevalence was reported in police officers after the WTC attack (5.4%) [8], and in the London Ambulance Service after the London bombings (6%) [21]. These terror attacks represent different circumstances: in London three underground stations and a bus were attacked; in Oklahoma and New York City, buildings were destroyed. Many peers were injured and some killed especially in the 9/11 attacks, but in both events rescue personnel were working inside of collapsing constructs, with dust, fire and dangerous substances. In Madrid, four trains were bombed, despite of this the sites were perhaps more secure and comprehensible being in daylight. In Norway one terror site was cleared before the next was attacked. In addition there were sufficient recourses and personnel. The great support from politicians and the general population may have been rewarding and reflected by the low prevalence of PTSS. In addition, the first responders were only involved in actions that are socially accepted and valued.

Previous studies have reported a higher level of distress and PTSS among ambulance personnel compared with other groups of rescue workers [4]. It has been hypothesized that this may be related to greater pressure and stress in their everyday work settings, and closer contact with victims, which may foster a process of identification. In our study, however, there were no significant differences between the groups in terms of PTSS. The higher prevalence in studies of everyday work-stress may be explained by ambulance personnel handling seriously injured and dead victims after e.g. motor vehicle accidents while the police officers working at the same site are securing traffic etc. (more distant). In an ongoing terror act, the circumstances are more similar between the groups of first responders.

Some studies have questioned whether a more inclusive and dimensional conceptualization of PTSD is required, particularly for rescue workers, because operational definitions and conventional screening cut-off points may underestimate the psychological burden of this population. Thus, even if there is a low prevalence of full PTSD, there are more subjects with stress related symptoms and functional impairment that may call for professional help.

### Predictors of PTSS

Dissociation was an independent predictor of PTSS in all groups, which agrees with a previous overview [22]. Witnessing injured victims was another independent predictor of PTSS in ambulance personnel. Ambulance personnel’s primary work task was to perform medical first aid, and thus, both witnessing serious injuries, and having physical contact with injured people. This may trigger symptoms of dissociation. A high proportion of fire-fighters reported to have physical contact with deceased victims, and perhaps a need for psychological distance. This may be one explanation of dissociation as an independent predictor in this group.

Feeling overwhelmed was an independent predictor of PTSS in police officers. Lack of resources, equipment, and personnel during rescue operations are risk factors for PTSD [6]. Equipment and personnel were available during the terror events in Norway, which indicates that the first responders were able to help those who were in need. The police officers reported to have less resources compared to the other groups. Ongoing shooting, and challenges for the police at Utøya Island may explain a feeling of being overwhelmed. The police received some criticism after the events, whereas the ambulance personnel and fire-fighters generally were praised. This may have influenced the responses from the police officers.

### Strengths and limitations

The response rates were moderate to very good (51 – 82%). Even though we cannot know the true response rate, we feel confidant that it is satisfactory, and that our main conclusions are valid.

Some of the instruments were designed specifically for this study, whereas most were developed by the Norwegian Centre of Violence and Traumatic Stress Studies, and are published elsewhere. The use of a validated questionnaire such as the PCL-S was a strength. Conducting interviews, which might yield additional information, would strengthen the study, but this would require considerable investment. However, it is unlikely that this would have changed our main finding that the level of posttraumatic stress was low.

Men dominated the professional groups involved in our study, which may be associated with a tendency to underreport symptoms that are not considered to be “masculine.” This may indicate that the reported levels of the symptoms are minimum values. People on sick leave might not have received the questionnaires, which may have biased the results. Data not presented here, show a low rate of sick leave. It is possible that some of the non-responders were on sick leave, which may have biased towards a slight underestimation of the PTSS level. However, the level of PTSS was very low, so it is unlikely that this would have been a major bias. We did not perform assessments of coping styles, personality traits, or previous or current psychiatric problems. In addition, we did not measure marital status or educational levels of the participants, or whether they had lost someone close during the attacks.

The participants completed the questionnaire only once, at 8–11 months after the event. Time can be a significant moderator of predictors. A meta-analysis of the predictors of PTSD [9] showed that the average effect size was greater for two predictors; life threat and peritraumatic dissociation, in studies where six months to three years had elapsed since the trauma. We only had one measurement point and were unable to study changes over time. It is likely that the levels of symptoms would have been higher shortly after the event. Thus, it may be considered a weakness of this study that we did not conduct a prospective design with at least two time points. On the other hand, a prospective design could not be anonymous, and would probably have lowered the response rate. By determining the symptoms after almost one year, we obtained data that demonstrated the long-term effects of the event.

### Clinical implications

The low rates of possible PTSD and PTSS indicate that the first responders who participated were quite resilient. The low levels of stress after such a serious event may also indicate that preparedness, training, leadership with clear roles as well as peer-support have been functioning. These factors should be studied in future research. Rescue workers with symptoms of distress should be identified, primarily by leaders and colleagues to get support and if necessary mental health services.

## Conclusions

First responders who participated in the rescue operations on July 22, 2011 were exposed to deaths, injuries, and destruction, but very few reported that this was a highly stressful experience. They reported low levels of dissociation during the events, but symptoms of dissociation were found to be the most important predictors of posttraumatic stress symptoms. The prevalence of possible PTSD was very low compared with most results reported in previous studies of terror attacks.

## References

[1] Sollid SJ, Rimstad R, Rehn M, Nakstad AR, Tomlinson AE, Strand T, Sandberg M (2012). Oslo government district bombing and utoya island shooting July 22, 2011: the immediate prehospital emergency medical service response. Scand J Trauma Resusc Emerg Med.

[2] Gaarder C, Jorgensen J, Kolstadbraaten KM, Isaksen KS, Skattum J, Rimstad R, Naess PA (2012). The twin terrorist attacks in Norway on July 22, 2011: The trauma centre response. J Trauma Acute Care Surg.

[3] Waage S, Poole JC, Thorgersen EB (2013). Rural hospital mass casualty response to a terrorist shooting spree. Br J Surg.

[4] Berger W, Coutinho ES, Figueira I, Marques-Portella C, Luz MP, Neylan TC, Mendlowicz MV (2012). Rescuers at risk: A systematic review and meta-regression analysis of the worldwide current prevalence and correlates of PTSD in rescue workers. Soc Psychiatry Psychiatr Epidemiol.

[5] Norris FH, Friedman MJ, Watson PJ, Byrne CM, Diaz E, Kaniasty K (2002). 60,000 disaster victims speak: Part I. An empirical review of the empirical literature, 1981. 2001. Psychiatry.

[6] Declercq F, Meganck R, Deheegher J, Van HH (2011). Frequency of and subjective response to critical incidents in the prediction of PTSD in emergency personnel. J Trauma Stress.

[7] Ozer EJ, Best SR, Lipsey TL, Weiss DS (2003). Predictors of posttraumatic stress disorder and symptoms in adults: a meta-analysis. Psychol Bull.

[8] Pietrzak RH, Schechter CB, Bromet EJ, Katz CL, Reissman DB, Ozbay F, Southwick M (2012). The burden of full and subsyndromal posttraumatic stress disorder among police involved in the World Trade Centre rescue and recovery effort. J Psychiatr Res.

[9] Meyer EC, Zimering R, Daly E, Knight J, Kamholz BW, Gulliver SB (2012). Predictors of posttraumatic stress disorder and other psychological symptoms in trauma exposed fire-fighters. Psychol Serv.

[10] Jonsson A, Segesten K, Mattsson B (2003). Post-traumatic stress among Swedish ambulance personnel. Emerg Med J.

[11] Lilly MM, Pole N, Best SR, Metzler T, Marmar CR (2009). Gender and PTSD: what can we learn from female police officers?. J Anxiety Disord.

[12] Thoresen S, Tonnessen A, Lindgaard CV, Andreassen AL, Weisaeth L (2009). Stressful but rewarding: Norwegian personnel mobilised for the 2004 tsunami disaster. Disasters.

[13] Weathers F, Litz B, Herman DS, Huska J, Keane TM (1993). The PTSD checklist (PCL): handbook of PTSD. Science and Practice.

[14] Elhai JD, Gray MJ, Kashdan TB, Franklin CL (2005). Which instruments are most commonly used to assess traumatic event exposure and posttraumatic effects?: A survey of traumatic stress professionals. J Trauma Stress.

[15] American Psychiatric Association (APA) (1994). Diagnostic and statistical manual of mental disorders.

[16] Yeager DE, Magruder KM, Knapp RG, Nicholas JS, Frueh C (2007). Performance characteristics of the posttraumatic stress disorder checklist and SPAN in veteran Affaires primary care settings. Gen Hosp Psychiatry.

[17] Harrington T, Newman E (2007). The psychometric utility of two self-report measures of PTSD among women substance users. Addict Behav.

[18] Gabriel R, Ferrando L, Cortòn ES, Mingote C, García-Camba E, Liria AF (2007). Psychopathological consequences after a terrorist attack: An epidemiological study among victims, the general population, and police officers. European Psychiatry.

[19] Stellman JM, Smith RP, Katz CL, Sharma V, Charney DS, Herbert R, Southwick S (2008). Enduring mental health morbidity and social function impairment in world trade centre rescue, recovery, and cleanup workers: the psychological dimension of an environmental health disaster. Environ Health Perspect.

[20] North CS, Tivis L, McMillen JC, Pfefferbaum B, Spitznagel EL, Cox J, Smith EM (2002). Psychiatric disorders in rescue workers after the Oklahoma City bombing. Am J Psychiatr.

[21] Misra M, Greenberg N, Hutchinson C, Brain A, Glozier N (2009). Psychological impact upon London ambulance service of the 2005 bombings. Occup Med.

[22] Marmar CR, McCaslin SE, Metzler TJ, Best S, Weiss DS, Fagan J, Neyland T (2006). Predictors of posttraumatic stress in police and other first responders. Ann N Y Acad Sci.

